# Ly6G+ neutrophil-derived miR-223 inhibits the NLRP3 inflammasome in mitochondrial DAMP-induced acute lung injury

**DOI:** 10.1038/cddis.2017.549

**Published:** 2017-11-16

**Authors:** Zunyong Feng, Shimei Qi, Yue Zhang, Zhilin Qi, Liang Yan, Jing Zhou, Fang He, Qianqian Li, Yanyan Yang, Qun Chen, Shi Xiao, Qiang Li, Yang Chen, Yao Zhang

**Affiliations:** 1 Anhui Province Key Laboratory of Active Biological Macro-molecules, Wannan Medical College, Wuhu, China; 2 Department of Forensic Medicine, Wannan Medical College, Wuhu, China; 3 Department of Biochemistry, Wannan Medical College, Wuhu, China; 4 Department of Intensive Care Unit, Affiliated Yijishan Hospital of Wannan Medical College, Wuhu, China

## Abstract

MicroRNA (miRNA) mediates RNA interference to regulate a variety of innate immune processes, but how miRNAs coordinate the mechanisms underlying acute lung injury/acute respiratory distress syndrome (ALI/ARDS) in patients with pulmonary inflammatory injury is still unknown. In this study, we demonstrated that miR-223 limits the number of Ly6G+ neutrophils and inhibits the activity of the NLRP3 inflammasome to alleviate ALI induced by mitochondrial damage-associated molecular patterns (DAMPs) (MTDs). miR-223 expression is increased in the lungs of MTD-induced mice or ARDS patients following trauma/transfusion or following the physiological remission of ALI/ARDS. miR-223−/+ mice exhibited more severe ALI and cytokine dysregulation. Other studies have shown that MTD-induced increases in miR-223 expression are mainly contributed by Ly6G+ neutrophils from the haematopoietic system. miR-223 blocks bone marrow-derived Ly6G+ neutrophil differentiation and inhibits peripheral cytokine release. In addition, MTD-induced miR-223 expression activates a negative feedback pathway that targets the inhibition of NLRP3 expression and IL-1*β* release; therefore, miR-223 deficiency can lead to the sustained activation of NLRP3-IL-1*β*. Finally, elimination of peripheral Ly6G+ neutrophils and pharmacological blockade of the miR-223–NLRP3–IL-1*β* signalling axis could alleviate MTD-induced ALI. In summary, miR-223 is essential for regulating the pathogenesis of DAMP-induced ALI.

Acute lung injury/acute respiratory distress syndrome (ALI/ARDS) is a respiratory disease characterized by diffuse alveolar injury, oedema, bleeding, the formation of a transparent membrane and polymorphonuclear neutrophil infiltration.^1^ ARDS is a more serious form of ALI and is characterized by a rapid onset, poor prognosis and high mortality; it is one of the major causes of death in intensive care units worldwide.^2, 3, 4, 5, 6^ Although the clinical risk factors of ARDS has been elucidated, it is difficult for clinicians to assess the clinical risk factors and predict the survival and progression of ARDS. The pathology of ARDS, especially non-pulmonary ARDS (caused by trauma, blood transfusion and transplantation), is still difficult to articulate as it involves complex genetic, environmental and immune interactions.

Pattern recognition receptor (PRR) is a membrane-bound or cytoplasmic receptor that recognizes exogenous components known as pathogen-associated molecular patterns (PAMPs) and endogenous damage-associated molecular patterns (DAMPs). PRR is essential for innate immunity. Toll-like receptors (TLRs) function as membrane-bound immune PRRs, and a variety of functional human TLRs have been identified.^7^ The development of infectious or pulmonary ALI/ARDS is related to activation of the TLR signalling pathway. Hyaluronic acid produced by tissue injury is mediated by TLR2/4, which contributes to the development of ARDS.^8^ Endotoxin release from Gram-negative bacteria is mediated by TLR4.^9^ In addition, TLR3 mediates hyperoxia-induced ARDS,^10^ whereas TLR2 mediates bleeding-induced ARDS.^11^ In addition to membrane-bound PRRs, the PRRs in the cytoplasm, such as the NLRP3 inflammasome, can recognize PAMPs and DAMPs indirectly. This process can be referred to as the detection of homeostasis-altering molecular processes. The NLRP3 inflammasome is composed of NLRP3, ASC and caspase-1. When NLRP3 inflammasomes recognize PAMPs and DAMPs, caspase-1 cleaves pro-IL-1*β* and pro-IL-18, producing active IL-1*β* and IL-18, thereby mediating multiple inflammatory responses.^12, 13^

Studies have shown that NLRP3 is highly associated with trauma- and transfusion-induced ARDS, and traumatic/surgical injuries release tissue-derived mitochondrial DAMPs (MTDs) into circulation to activate NLRP3 inflammasomes in neutrophils.^14^ Transfusion also produces circulating MTDs.^15^ MTDs include mitochondrial DNA, formyl peptides and cardiolipin, which induce DAMPs during lung endothelial cell injury.^16, 17, 18^ Mitochondria-targeted inhibitors have been shown to reduce DAMP-induced damage in mice mouse lung endothelial cells.^19^ MTDs may be a direct cause of aseptic inflammation and clinical symptoms in non-pulmonary ARDS.^20^ Therefore, research on MTD-mediated injury is necessary for the development of non-pulmonary ARDS awareness and treatment strategies.

MicroRNAs (miRNAs) have a vital role in the maintenance of immune homeostasis.^21, 22, 23^ However, the regulation of miRNA in ARDS also lacks effective understanding. miRNA-223 was the first miRNA to be specifically identified in the haematopoietic system,^24^ and it regulates natural immune homeostasis and *Mycobacterium tuberculosis*-induced stress responses.^21, 25^ miR-223 is induced during myeloid differentiation and is regulated by a variety of genes.^24^ In addition, miR-223 regulates NF-*κ*B-dependent macrophage differentiation,^26^ E2F1-dependent granulocyte differentiation,^27^ inflammation, infection and tumour progression.^28^ Several experiments have demonstrated that miR-223 represses NLRP3 inflammatory bodies; however, the role of miR-223 in NLRP3-mediated non-pulmonary ARDS has not been studied.

## Results

### miR-223 participates in MTD-induced ARDS

**Figure 1:**
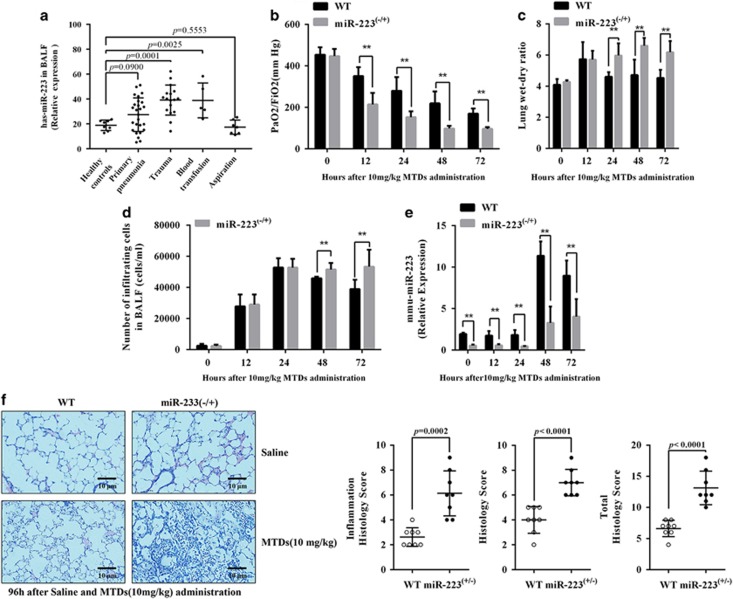
The correlation between miR-223 and ARDS. (**a**) miR-223 levels in BALF from ARDS patients with different risk factors (*n*=5–26). (**b**) The PaO_2_/FiO_2_ (*n*=12) and (**c**) the number of inflammatory cells in BALF were measured at 0, 12, 24, 48 and 72 h after the administration of 10 mg/kg MTDs to (**d**) WT and miR-223−/+ mice (*n*=6). WT and miR-223−/+ mice were administered MTDs, and (**c**) the lung dry/wet weight ratio (*n*=6) and (**e**) the pulmonary mmu-miR-223 level (*n*=6) were measured after 0, 12, 24, 48 and 72 h. The WT and miR-223−/+ mice were administered MTDs for 96 h, and the lung tissue was paraffin embedded, sliced and HE stained to assess inflammatory cell infiltration and lung injury based on the following scores: mild (0–4), mild (5–6), moderate (7–8), and severe (9–10). The total score=inflammation score+injury score (*n*=8) (× 400 magnification). All data are presented as the mean±S.E.M., and comparisons between groups were performed using *t*-tests. *P<0.05 and **P<0.01 *versus* the WT group

We screened patients with acute ARDS based on the Berlin definition^29^ and selected those with ARDS caused by primary pneumonia, trauma, blood transfusion and inhalation. A total of 29 miRNAs that are highly associated with the inflammatory response were detected as described by Chen *et al.*^30^ As shown in Supplementary Figure S1A, miR-223 expression was the highest in bronchoalveolar lavage fluid (BALF) from patients with ARDS caused by trauma and blood transfusion. qPCR was used to detect the expression level of miR-223. The results are shown in Figure 1a. The expression of miR-223 of patients with ARDS caused by trauma and blood transfusion was significantly upregulated, whereas ARDS caused by infection and inhalation remained unchanged. In addition, we analysed the relationship between miR-223 levels from the acute period of ARDS and ΔPaO_2_/FiO_2_, using the PaO_2_/FiO_2_ difference before and after 4 days of positive end-expiratory pressure (PEEP) treatment as a prognostic index. The results are shown in Supplementary Figure S1B. There is a linear relationship between has-miR-223 and ΔPaO_2_/FiO_2_ compared with the control group. In other words, routine treatment will be more effective among ARDS patients with higher has-miR-223 expression.

The mouse model of ARDS was induced by an intravenous injection of MTDs, and the results are shown in Figures 1b–d. Following the induction by MTDs, the PaO_2_/FiO_2_ of mice was significantly decreased, the proportion of lung wet weight was increased and the number of inflammatory cells in the lung was increased. Compared with the wild-type (WT) group, miR-223−/+ mice exhibited more serious MTD-induced lung injury; thus the recovery from lung injury was delayed. Figure 1e shows that miR-223 expression began to increase in WT mice 48 h after MTD stimulation, which was later than the induction of ALI by MTDs. miR-223 expression in miR-223−/+ mice was lower than that in WT mice and had no obvious effect on MTDs. Histological results, as shown in Figure 1f, indicate that the lung injury and inflammation in the miR-223−/+ mouse lungs was greater than that in the WT mice 4 days after induction by MTDs. This evidence suggests that miR-223 deficiency aggravates MTD-induced ALI.

### Haematopoietic cell-derived miR-223 relieves MTD-induced ALI

**Figure 2:**
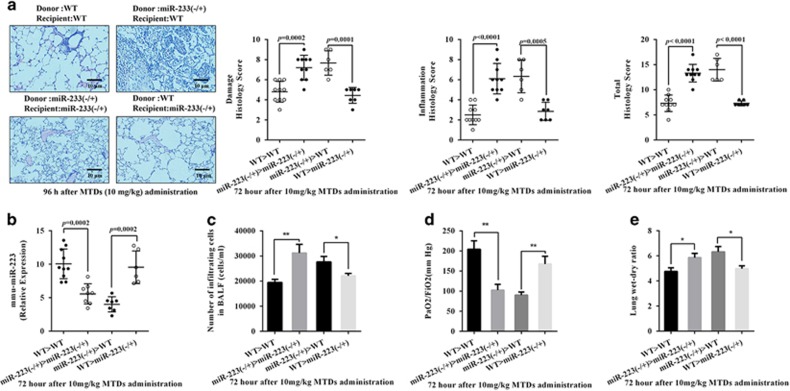
miR-223 expression is contributed by haematopoietic cells. After excessive exposure to X-ray, WT and miR-223−/+ mice were immediately transplanted with BM cells from miR-223−/+ and WT mice, respectively, thus producing BM chimeric mice. (**a**) The inflammatory score and injury score were assessed in WT, miR-223−/+ and BM chimeric mice 96 h after the administration of MTDs. (**b**) The PaO_2_/FiO_2_, (**c**) lung dry/wet weight ratio, (**d**) lung mmu-miR-223 level and (**e**) number of inflammatory cells in BALF were measured. All data are presented as the mean±S.E.M., and comparisons between groups were performed using *t*-tests. *P<0.05 and **P<0.01; *n*=6–10

In this study, the miR-223−/+ mice were knockout heterozygous mice, and it is unknown whether miR-223 deficiency in the lung or blood causes the increase in lung injury. To solve this problem, we constructed bone marrow (BM) chimeric mice lung injury model. The results are shown in Figure 2a. Both WT and miR-223−/+ mice that received BM cells from miR-223−/+ mice were sensitive to MTD-induced lung injury after excessive exposure to X-ray, whereas BM cells from WT mice were insensitive to MTD induction. This indicated that MTD-induced lung injury is mediated by the haematopoietic system. In addition, WT and miR-223−/+ mice acquired the opposite phenotype after transplantation with the other’s BM, including the PaO_2_/FiO_2_ (Figure 2d), lung wet/dry weight ratio (Figure 2e) and the number of inflammatory cells in the lungs (Figure 2c). Meanwhile, Figure 2b shows that miR-223 expression in WT mice that received miR-223−/+ mouse BM cells was significantly lower than that in miR-223−/+ mice transplanted with WT mouse BM cells. These results indicate that miR-223 deficiency in haematopoietic-derived cells leads to aggravated MTD-induced lung injury in mice.

### miR-223 from Ly6G+ neutrophils

**Figure 3:**
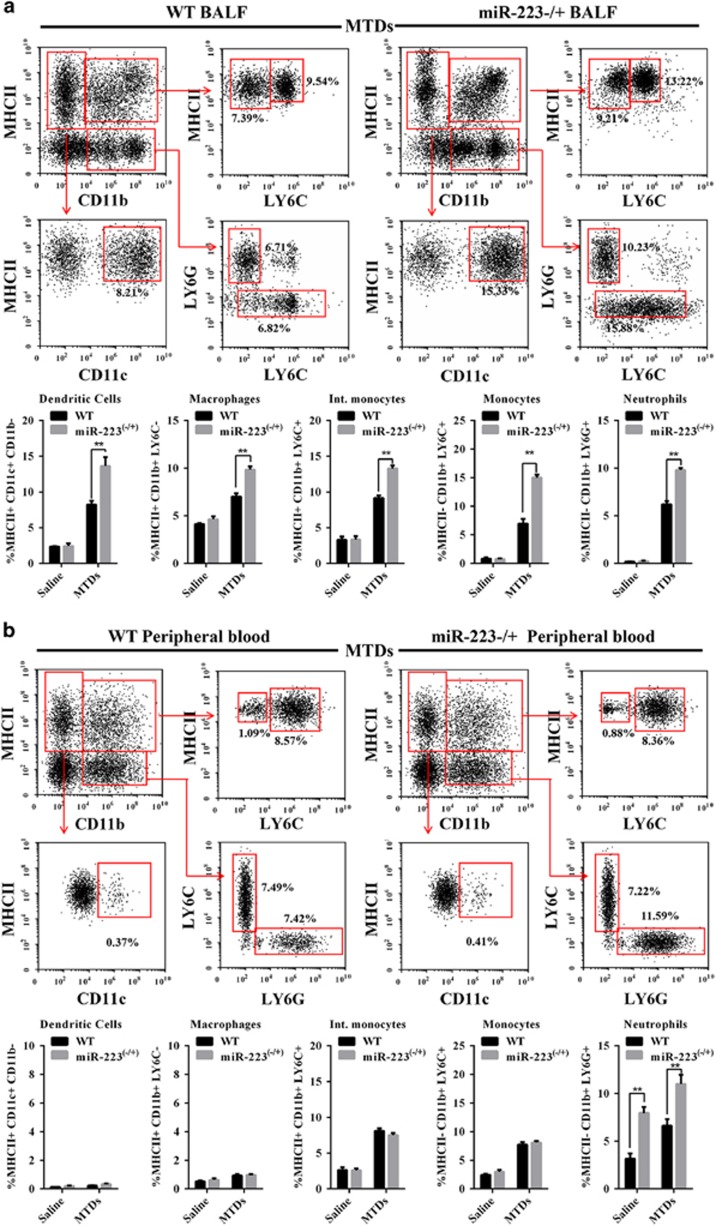

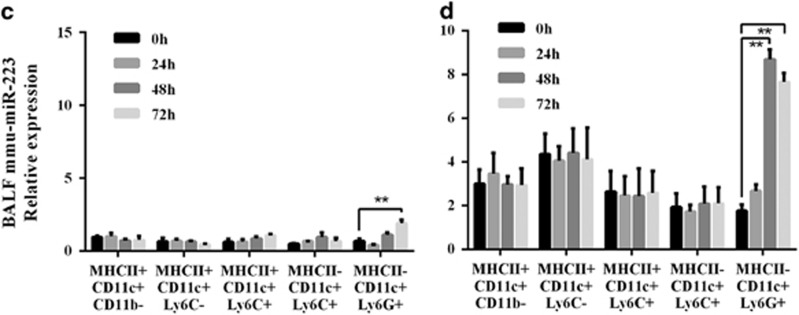
Cell populations in BALF and peripheral blood. WT and miR-223−/+ (*n*=6) mouse (**a**) BALF and (**b**) peripheral blood were collected 48 h after the administration of MTDs. After labelling with specific antibodies, including MHCII, CD11b, LY6C, LY6G and CD11c, flow cytometry was performed to identify neutrophils (Ly6G+ MHCII−), monocytes (Ly6C+ MHCII−), intermediate monocytes (Ly6C+ MHCII+), macrophages (Ly6C− MHCII+) and dendritic cells (MHCII+ CD11c+ CD11b−) (*n*=6). At 12, 24 and 48 h after the administration of MTDs (*n*=3), (**c**) miR-223−/+ and (**d**) WT mouse myeloid cells were sorted, and the expression of mmu-miR-223 was detected by TaqMan. All data are presented as the mean±S.E.M., and comparisons between groups were performed using *t*-tests. *P<0.05 and **P<0.01; *n*=3–6

The proportion of myeloid cells in the BALF of ARDS patients was detected. As shown in Supplementary Figures S2A and B, the proportion of LY6G+ neutrophils and LY6C+ monocytes in ARDS patients during the acute period was significantly increased. To identify the major source of miR-223, we examined the myeloid cell population in WT and miR-223−/+ mouse BALF. The results are shown in Figure 3a. Three days after MTD induction, the myeloid cells was significantly increased in miR-223−/+ mice compared with WT mice. Figure 3b shows that induction with MTDs resulted in increased myeloid cell populations in the peripheral blood of miR-223−/+ and WT mice. It is worth noting that, both at the physiological level and MTD-induced level, the Ly6G+ cells in the peripheral blood of miR-223−/+ mice were significantly higher than that in WT mice. Therefore, miR-223-deficiency leads to an increase in Ly6G+ cells in the peripheral blood of mice. Flow cytometry was used to sort the myeloid cells, and miR-223 were detected by qPCR. The results, which are shown in Figure 3c, indicate that miR-223 was highly expressed in the Ly6G+ cells of WT mice at levels greater than in miR-223−/+ mice after stimulation by MTDs. These results suggest that the increase in miR-223 expression following MTD stimulation is contributed by Ly6G+ cells from the haematopoietic system.

### miR-223 blocks granulocyte and monocyte precursors from differentiating into LY6G+ cells

**Figure 4:**
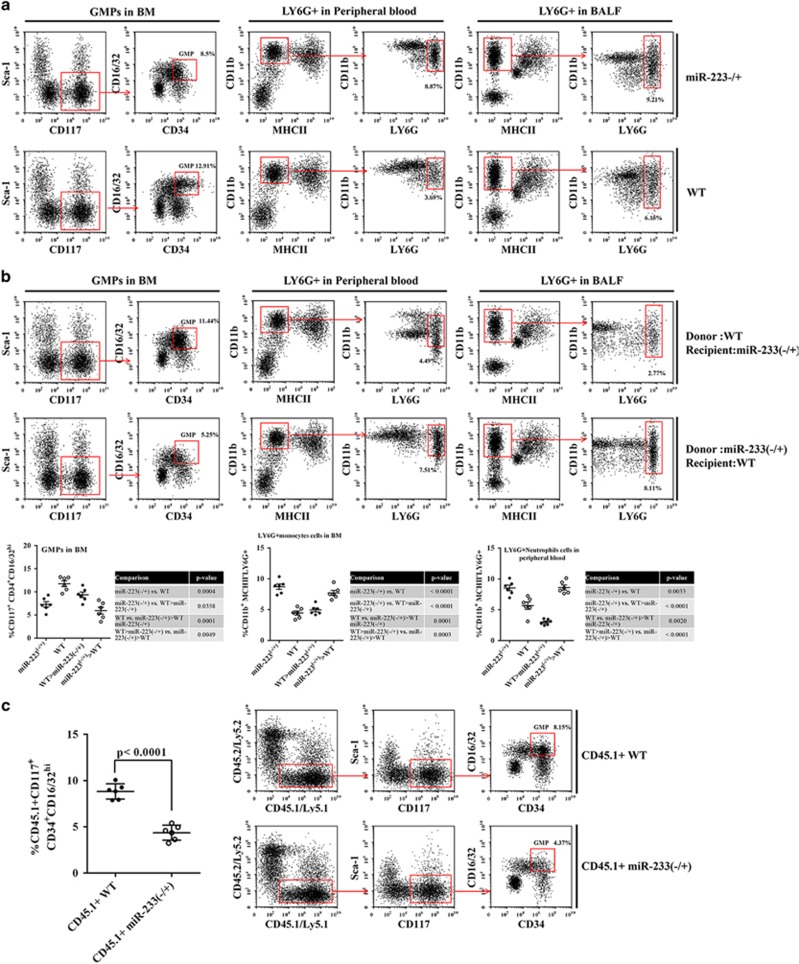
Correlation between miR-223 and GMP differentiation into LY6C+ cells. (**a**) WT and miR-223−/+ mice were treated with MTDs; after 48 h, the percentage of GMPs (Sca-1− CD117+ CD34+ CD16/32^hi^) in the BM and the percentage of neutrophils (CD11b+ Ly6C− Ly6G+) in the peripheral blood and lungs were measured by flow cytometry. (**b**) BM chimeric mice were treated with MTDs, and after 48 h, the percentage of GMPs in the bone marrow and the neutrophils in the peripheral blood and lungs were measured. CD45.1+ WT or CD45.1+ miR-223−/+ mouse BM cells were transferred together with an equal number of CD45.2+ WT mouse BM cells into lethally irradiated CD45.2+ WT mouse recipients. (**c**) Then, 2 weeks after transplantation, GMPs (CD45.1+ Sca-1− CD117+ CD34+ CD16/32^hi^) derived from BM cells of CD45.1+ miR-223−/+ mice were observed. All data are presented as the mean±S.E.M., and comparisons between groups were performed using *t*-tests. *P<0.05 and **P<0.01; *n*=6

Peripheral LY6G+ cells are mainly differentiated from BM granulocyte and monocyte precursors (GMPs).^31^ To investigate the effect of miR-223 on the number of LY6G+ cells, the effect of miR-223 on the differentiation of GMPs to Ly6G+ cells was examined. As shown in Figure 4a, the number of GMPs in the miR-223−/+ mouse BM and the number of Ly6G+ cells in the peripheral blood and BALF were significantly higher and lower than those in WT mice, respectively. The percentage of Ly6G+ cells in the peripheral blood and BALF of WT mice transplanted with miR-223−/+ mouse BM cells and miR-223−/+ mice transplanted with WT mouse BM cells was also increased and decreased, respectively. Therefore, WT mice and miR-223−/+ mice transplanted with the other’s BM cells exhibited the opposite myeloid cell characteristics, suggesting that miR-223 inhibits the differentiation of GMPs into Ly6G+ cells.

To determine whether GMP differentiation to Ly6G+ cells is affected by the intrinsic or extrinsic need for miR-223, we generated BM chimeric mice in which CD45.1+ WT or CD45.1+ miR-223−/+ BM cells were transferred with an equal number of CD45.2+ BM cells into lethally irradiated CD45.2+ recipients. Then, 2 weeks after transplantation, we observed significant reductions in the frequency of GMPs derived from BM cells of CD45.1+miR-223−/+ mice (Figure 3c), indicating a cell-intrinsic defect in the differentiation of GMPs in miR-223-deficient mice. This suggests that the differentiation of GMPs into Ly6G+ cells is caused by the increased endogenous expression of miR-223 in GMP cells.

### miR-223-deficiency aggravates MTD-induced cytokine dysregulation

**Figure 5:**
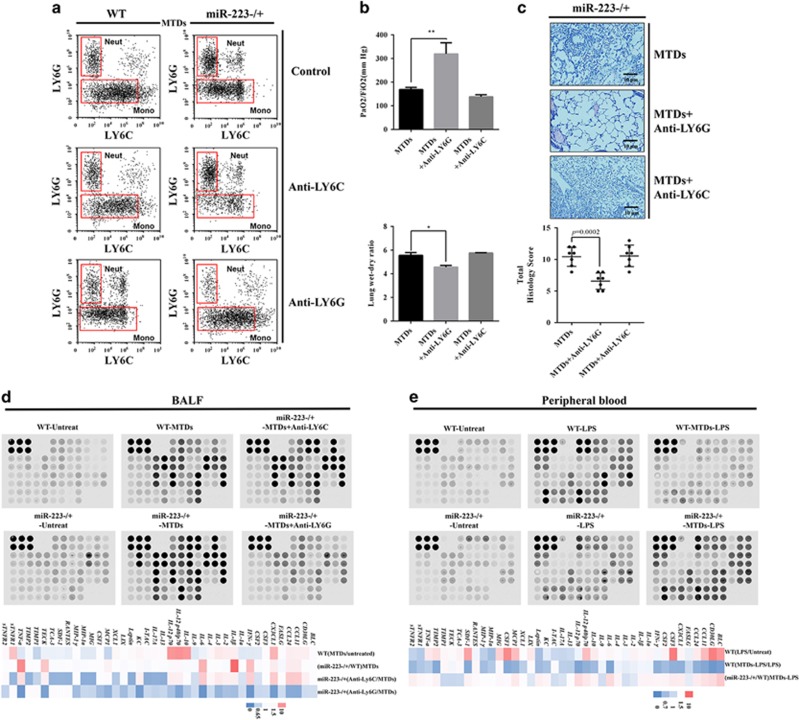
LY6G+ neutrophils mediate MTD-induced ALI and cytokine release. WT and miR-223−/+ mice were treated with anti-Ly6G (300 *μ*g/day, i.p.) or anti-Ly6C (200 *μ*g/day, i.p.) for 5 consecutive days, and (**a**) flow cytometry was used to detect MHCII, LY6C and LY6G cell surface antigens. miR-223−/+ mice were treated with anti-Ly6G or anti-Ly6C 48 h after the administration of MTDs. (**b**) Then the PaO_2_/FiO_2_ and lung dry/wet ratio were measured and (**c**) the pathological score was assessed (*n*=7). WT and miR-223−/+ mice were treated with MTDs, and after 24 h. (**d**) The relative levels of 40 inflammatory cytokines and chemokines in BALF were detected in the BALF of MTD- and anti-Ly6G- or anti-Ly6C-treated mice with a microarray chip (*n*=3). Twenty-four hours after WT and miR-223−/+ mice were treated with MTDs, they were treated with 5 mg/kg lipopolysaccharide (LPS). (**e**) After another 24 h, the cytokine levels were detected (*n*=3). All data are presented as the mean±S.E.M., and comparisons between groups were performed using *t*-tests. *P<0.05 and **P<0.01

To verify the role of Ly6G+ cells in mediating lung injury following MTD induction, we used antibodies to eliminate Ly6G+ and Ly6C+ cells in the peripheral blood (Figure 5a) and subsequently evaluated MTD-induced ALI in mice. The results are shown in Figures 5b and c. Treatment with Ly6C antibody did not have beneficial effects on MTD-induced lung injury, whereas Ly6G antibody significantly improved lung injury. The clinical data also show that there is a linear relationship between LY6G+ cells and the ΔPaO_2_/FiO_2_ in ARDS patients (Supplementary Figure S1C), where higher proportions of LY6G+ cells in the lungs of ARDS patients during the acute period correlate with worse prognosis following conventional therapy. At the same time, the linear relationship between miR-223 and LY6G+ cells (Figure 1e) showed that miR-223 was negatively correlated with the percentage of LY6G+ cells in the lungs of ARDS patients. These evidence fully demonstrates that Ly6G+ cells mediate MTD-induced damages to the lung tissue.

Next, we examined the release of various cytokines in the lungs. As shown in Figure 5d, miR-223-deficient mice exhibited more severe inflammatory cytokine and chemokine dysregulation compared with WT mice, especially in the expression of IL-1*β*, IL-6 and TNF-*α*. In addition, the release of cytokines was detected after using Ly6C and Ly6G antibodies, respectively. The Ly6G antibody could ameliorate the MTD-induced cytokine dysregulation, but the Ly6C antibody could not ameliorate the dysregulation. Studies have shown that patients or rats with ARDS caused by trauma or surgery exhibit systemic immunosuppression.^32, 33^ Thus we investigated whether systemic immunosuppression of non-pulmonary ARDS was regulated by miR-223. The results are shown in Figure 5e. Cytokine release in MTD-induced WT mice was significantly inhibited by lipopolysaccharide stimulation, whereas cytokines in the peripheral blood of miR-223−/+ mice was significantly greater than that in the WT mice, indicating that miR-223 was a possible cause of peripheral immunosuppression in non-pulmonary ARDS. However, additional investigation is required to confirm this finding.

### miR-223 negative feedback regulation inhibits NLRP3 expression in MTD-induced BM-derived neutrophils

**Figure 6:**
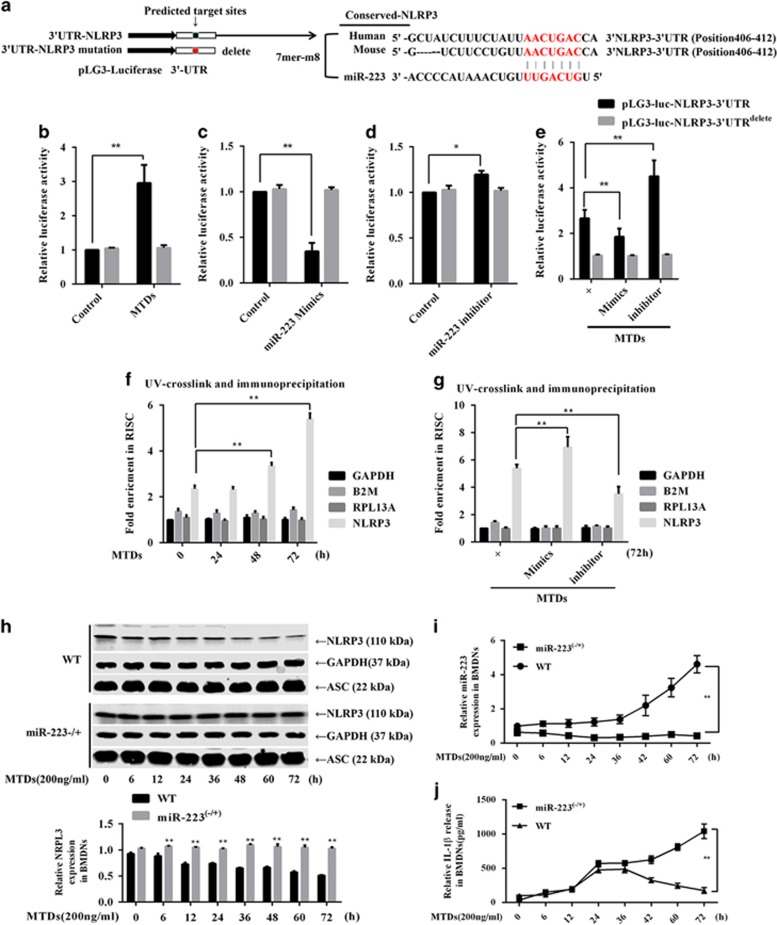
miR-223 inhibits NLRP3 expression. (**a**) The pGL3-luc-NLRP3 3′UTR luciferase reporter construct and deletion of the predicted miR-223-binding site in the 3′UTR. WT BMDNs were sorted. NLRP3-3′UTR or NLRP3-3′UTR^delete^ luciferase plasmids were transfected into BMDNs. (**b**) Then they were treated with 200 ng/ml MTDs for 24 h, and the luciferase activity in the BMDNs was detected. miR-223 (**c**) mimics or (**d**) inhibitor was co-transfected with the NLRP3 3′UTR reporter plasmids into BMDNs. (**e**) miR-223 mimics or inhibitor was co-transfected with the luciferase plasmids into BMDNs, which were then treated with MTDs for 24 h. BMDNs were treated with (**f**) MTDs for 24, 48 and 72 h and (**g**) miR-223 mimics or inhibitor for 72 h; for the PAR-CLIP experiment, the co-precipitated NLRP3 mRNA levels were detected by quantitative PCR (qPCR); GAPDH, B2M and RPL13 mRNA was also detected. WT and miR-223−/+ mouse BMDNs were sorted, and MTDs were used to stimulate the cells for varying amounts of time. Western blotting and qPCR were used to detect the expression of (**h**) NLRP3 and (**i**) miR-223, and (**j**) the release of interleukin-1*β* was detected by ELISA. All data are presented as the mean±S.E.M., and comparisons between groups were performed using *t*-tests. *P<0.05 and **P<0.01; *n*=3–6

IL-1*β* is mainly activated by the NLRP3 inflammasome, and we aimed to verify whether miR-223 inhibits the activation of NLRP3. The pGL3-luc-NLRP3 3′UTR luciferase reporter gene was constructed and the predicted 3′UTR-binding site was then deleted (Figure 6a). The NLRP3 3′UTR reporter was transfected into BM-derived neutrophils (BMDNs), then treated with 200 ng/ml MTDs for 6 h and the luciferase activity was detected. As shown in Figure 6b, MTDs upregulated the luciferase activity from the NLRP3 3′UTR reporter plasmid, whereas the NLRP3 3′UTR^delete^ plasmid was unchanged. miRNA-223 mimics or inhibitor was co-transfected into BMDNs with the NLRP3 3′UTR reporter plasmid, and the results are shown in Figures 6c and d. miR-223 mimics attenuates BMDN luciferase activity, whereas the inhibitor slightly upregulates BMDN luciferase activity. Meanwhile, the NLRP3 3′UTR^delete^ did not react to miR-223 mimics or inhibitor. In addition, Figure 6e indicates that the mimics and inhibitors attenuated and enhanced the MTD-induced NLRP3 3′UTR luciferase activity, respectively. After performing photoactivatable ribonucleoside-enhanced cross-linking immunoprecipitation (PAR-CLIP), qPCR was used to detect the level of NLRP3 mRNA that was co-precipitated. The results are shown in Figures 5f and g. MTDs could upregulate the enrichment of NLRP3 mRNA in RNA-induced silencing complex (RISC), whereas miR-223 mimics or inhibitors significantly upregulated or downregulated the accumulation of NLRP3 mRNA, respectively. These experiments show that MTDs can upregulate the translation of NLRP3 *in vitro*, whereas miRNA-223 can inhibit the translation of NLRP3 3′UTR in BMDNs.

Next, we examined the effect of MTDs on the level of NLRP3 and IL-1*β* in the BMDNs of WT and miR-223−/+ mice. The results are shown in Figures 6h–j. MTDs time-dependently upregulated miR-223 expression in WT mouse BMDNs and downregulated NLRP3 expression. In addition, in miR-223−/+ mouse BMDNs, NLRP3 expression was slightly changed in response to MTD induction, and correspondingly, IL-1*β* release was significantly greater than that in WT mouse. This evidence strongly suggests that miR-223 negative feedback regulates the expression of NLRP3 and that a miR-223 deficiency may cause NLRP3 inflammasome signal blockade in response to MTD stimulation, thus resulting in excessive production of IL-1*β*.

### Blocking NLRP3 and IL-1β alleviates MTD-induced ALI

**Figure 7:**
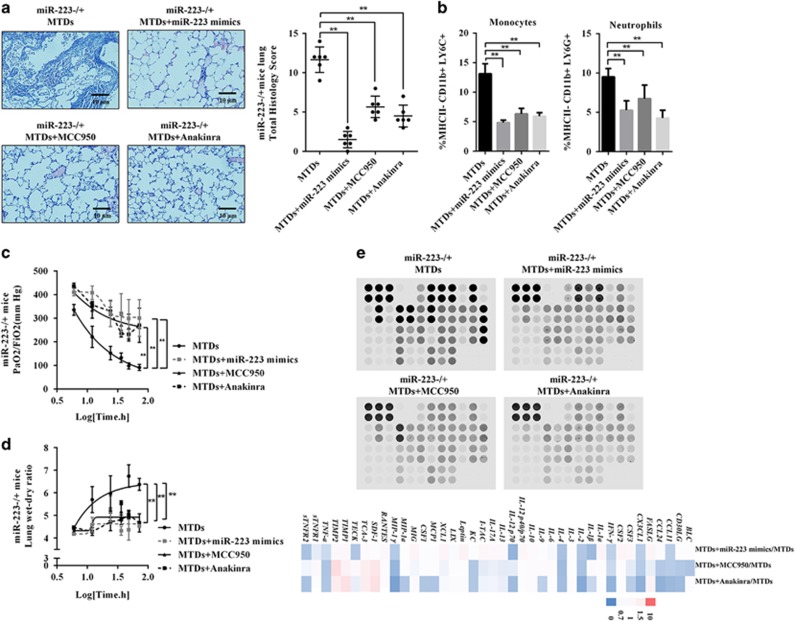
miR-223 mimics, MCC950 and anakinra treatment improved MTD-induced ALI. The NLRP3 small-molecule inhibitor MCC950 (20 mg/kg/day, i.p.), the interleukin-1R antagonist anakinra (20 mg/kg/day, i.p.) and miR-223 mimics (100 *μ*g/kg/day, i.v.) were administered to miR-223−/+ mice, which were immediately treated with MTDs. (**a**) HE staining was performed to evaluate the severity of the lung injury (× 400 magnification). The percentage of LY6C+ monocytes and LY6G+ neutrophils in (**b**) BALF, (**c**) PaO_2_/FiO_2_ and (**d**) lung dry/wet ratio were measured. (**e**) Cytokine levels in the miR-223−/+ mouse lungs induced by MTDs after treatment with MCC950, anakinra and miR-223 mimics were measured. All data are presented as the mean±S.E.M., and comparisons between groups were performed using *t*-tests. *P<0.05 and **P<0.01; *n*=3–6

Finally, we demonstrated whether selective blocking of the miR-223–NLRP3–IL-1*β* could alleviate MTD-induced ALI. miR-223−/+ mice were administered the NLRP3 small-molecule inhibitor MCC950, the IL-1R agonist anakinra or miR-223 mimics followed by the administration of MTDs. The results are shown in Figure 7a. MCC950, anakinra and miR-223 mimics all reduced MTD-induced ALI in miR-223−/+ mice. Figure 7b showed that Ly6C+ and Ly6G+ cells could be downregulated by MCC950, anakinra and miR-223 mimics treatment. In addition, PaO_2_/FiO_2_ was significantly decreased (Figure 7c), and the increase in the lung dry/wet weight ratio (Figure 7d) was reversed by the application of MCC950, anakinra and miR-223 mimics. More importantly, blocking miR-223–NLRP3–IL-1*β* could ameliorate the reduction of pulmonary cytokine release following MTD induction, as shown in Figure 7e. Clinical data also indicate the importance of IL-1*β* in the pathogenesis of ARDS. There was a negatively linear relationship between IL-1*β* and ΔPaO_2_/FiO_2_ or miR-223 in ARDS patients (Supplementary Figure S1C and F), where higher levels of IL-1*β* during the acute phase correlated with worse prognosis following conventional therapy. This evidence suggests that targeted molecular inhibition or pharmacological blockade of NLRP3 expression and IL-1*β* activity can effectively alleviate MTD-induced ALI.

## Discussion

Although ARDS occurs locally in the lungs, it is a systemic inflammatory disease involving both the lungs and other circulatory organs. Traumatic brain injury,^33^ sepsis^34^ and burns^35^ are risk factors for ARDS. Although the aetiology of non-pulmonary ARDS is still elusive (with genetic, environmental and immunological components), the general hypothesis suggests that ARDS is caused by a circulatory MTD-induced cytoplasmic PPR inflammatory response that leads to lung tissue damage. Cytoplasmic NLRP3 and IL-1*β* is a key regulator of the immune response to DAMP. miR-223 has been shown to inhibit NLRP3 expression *in vitro*, but its role in the NLRP3-mediated MTD-induced ALI process is unclear; therefore, understanding the molecular regulation of miRNA-223 during DAMP-induced injury may help improve the treatment of non-pulmonary ARDS.

In our study, miR-223 expression increased in the BALF of ARDS patients with non-infectious aetiologies (trauma and blood transfusion), and the correlation was significant. Although the miR-223 in patients with ARDS caused by infection and inhalation was not significantly different from that in the control patients, there was a positive linear relationship between the level of has-miR-223 in all patients with ARDS and the prognosis following conventional therapy. Thus, in this study, we focussed on the role of miR-223 in the pathogenesis of MTD-induced acute ARDS. *In vivo* experiments showed that MTDs induce hypoxemia, inflammatory and ALI in WT mice and the miR-223−/+ mice more sensitive to MTD induction. In addition, MTDs upregulate miR-223 expression, which occurs after the onset of the inflammatory response, and therefore, miR-223 may be used as a negative feedback molecule to alleviate MTD-induced ALI. We used X-rays to destroy the haematopoietic system of mice and found that WT and miR-223−/+ mice exhibited the opposite lung phenotype in response to MTD induction after transplantation of the other’s BM. BM chimeric mice showed that the haematopoietic system is the source of increased miR-223 expression.

Studies have shown that miR-223 is least expressed in tissue-infiltrated macrophages but highly expressed in circulating monocytes and neutrophils.^24, 36, 37, 38^ According to the clinical data we collected, miR-223 levels were positively correlated with LY6G+ cells in the lungs of patients with ARDS, whereas LY6G+ cells had a negative correlation with the prognosis of patients with ARDS, suggesting that LY6G+ cells may mediate the miR-223 mechanism of action. Therefore, we analysed the expression of miR-223 in the lung myeloid cell lineage. The results suggest that miR-223 deficiency leads to an increase in Ly6G+ cells in the peripheral blood of mice. Detection of miR-223 expression in the sorted cells revealed that the increase in miR-223 induced by MTDs was mainly contributed by Ly6G+ neutrophils, indicating that increased miR-223 will result in a decrease in the number of Ly6G+ neutrophils. Moreover, we examined the number of GMPs, which are the precursor population of Ly6G+ neutrophils, in the BM, and the results suggested that miR-223 blocks the differentiation of GMPs into LyG+ neutrophils. Furthermore, BM chimeric mouse experiments showed that miR-223 is essential for the differentiation of GMPs into Ly6G+ neutrophils. Further results showed that Ly6G antibody could block MTD-induced ALI and ARDS symptoms. These results suggest that miR-223 can lead to a decrease in the number of peripheral Ly6G+ neutrophils, thereby alleviating MTD-induced ALI.

Inflammatory cytokines, such as IL-1*β*, TNF-*α*, IL-6 and IL-8, are increased in the BALF of ARDS patients.^39^ Our clinical data show that the miR-223 is negatively correlated with serum IL-1*β* levels and that IL-1*β* levels are negatively correlated with the prognosis of ARDS patients. This suggests that downregulation of IL-1*β* mediates the beneficial effect of miR-223 on ARDS recovery. It is interesting to note that systemic immunosuppression has been observed in patients with non-pulmonary ARDS, and although there is a severe inflammatory response in the lungs, its potential underlying mechanism, according to this study, is presumed that miR-223 expression inhibits the number of Ly6G+ cells in the circulation and leads to feedback immunosuppression. The results suggest that systemic immunosuppression in patients with ARDS is dependent on the number of Ly6G+ cells and that MTDs induce the upregulation of miR-223 expression. In addition, MTDs significantly induce the expression of multiple inflammatory cytokines and chemokines in the lungs, and miR-223 deficiency leads to increased cytokine release. Ly6G antibodies can effectively block the MTD-induced release of cytokines, suggesting that Ly6G+ cells mediate a large amount of cytokine release.

miRNA-223 is a haematopoietic cell-specific miRNA, and recent studies have shown that miR-223 is almost exclusively expressed in myeloid cell lineage.^31^ miR-223 has a wide range of targeted inhibitory effects, including RHOB,^40^ FBXW7,^41^ FOXO^42^ and other important disease targets. *In vitro*, NLRP3 has been shown to be inhibited by miR-223^37^ and has received widespread attention. Nevertheless, the regulatory role of miR-223 in MTD-induced ARDS is still unknown. Our findings verified that miR-223 inhibited the expression of NLRP3 in BMDNs. In addition, the PAR-CLIP assay confirmed that the NLRP3 mRNA sequence was highly enriched in RISC after stimulation with MTDs and that the enrichment process was enhanced and attenuated by miR-223 mimics and inhibitors, respectively. More importantly, the decrease in NLRP3 expression in the BMDNs of WT mice is dependent on the upregulation of miR-223 and is time dependent after MTD stimulation. The expression of NLRP3 in the BMDNs of miR-223−/+ mice was not changed following MTD induction. Meanwhile, the level of IL-1*β* was consistent with the expression of NLRP3. This evidence suggests that miR-223 expression physiological feedback inhibits the MTD-induced activation of the NLRP3 inflammasome.

The current clinical treatments for ARDS include corticosteroid support therapy,^43^ recombinant colony-stimulating factor therapy,^44^ lipid metabolism interference,^45, 46^ lung surface active agent therapy,^47^ *β* agonist administration and NO inhalation.^48, 49^ However, only the use of neuromuscular blockers^50^ or mechanical ventilation^51^ can improve mortality. Therefore, there is a lack of effective treatments to address the cause of complex ALI/ARDS. Our study evaluated the effect of pharmacological blockade of miR-223–NLRP3–IL-1 on MTD-induced ALI, which resulted in better corrective efficacy. However, whether it can improve the mortality of patients with ARDS also requires further study.

**Figure 8:**
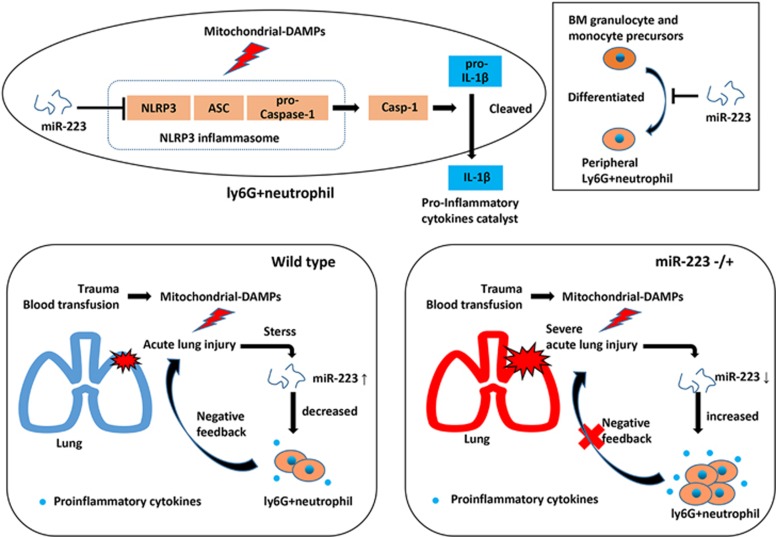
miR-223 is essential for regulating the pathogenesis of DAMP-induced ALI

In summary, our study highlights the key regulatory effect of Ly6G+ cells and the miR-223–NLRP3–IL-1*β* pathway in MTD-induced ARDS. As illustrated in Figure 8, miR-223 feedback inhibits NLRP3 activation, inhibits early pulmonary inflammatory factor dysregulation and relieves MTD-induced ALI. Thus genetic or pharmacological modulation of the miR-223–NLRP3–IL-1*β* pathway may be a promising new treatment for MTD-induced ALI and ARDS.

## Materials and methods

### Human subjects

BALF was collected from human subjects at the intensive care unit of the Wannan Medical College-affiliated Yijishan Hospital between January 2016 and March 2017. The Ethics Committee at the Yijishan Hospital of Wangnan Medical College approved all studies involving human subjects, and written informed consent was obtained from all patients. The experimental procedures for human subjects were strictly enforced based on the 1983 Declaration of Helsinki. Eligible ARDS patients were screened based on the Berlin definition.^29^ The basic data and risk factors of the patients are shown in Table 1. A total of 63 ARDS patients with different aetiologies and healthy controls were included: 42 were male and 21 were female. ARDS patients had refractory hypoxemia, severe dyspnoea and cyanosis; their respiratory rate was >29 breaths/min. The PaO_2_/FiO_2_, lung BALF and serum biochemical indicators were monitored in real time. All patients were given PEEP for 4 days as well as rehydration and antibiotic drug therapy. BALF or sub-lung level alveolar fluid was obtained by repeated sterile saline irrigation/lavage.

### Animals

miRNA-223−/+ mice and miRNA-223−/+ CD45.1+ mice on a C57BL/6J background were a gift from Dr. Bo Xu (Director of the Shaanxi Provincial People’s Hospital at the Third Affiliated Hospital of Xi’an Jiaotong University). C57BL/6J (CD45.2) mice and wild-type mice were purchased from Weitong Lihua Biotechnology (Beijing, China) (8–12-week old; 18–20 g, equal numbers of males and females) for subsequent experiments. Mice were housed in a specific-pathogen-free environment at a temperature of 22–25 °C with a relative humidity of approximately 40%, daily 12 h day/night alternation and free access to food and drink. Animal welfare and standard animal test procedures were strictly enforced based on the Guidelines for Laboratory Animal Care and Use (US National Research Council, 12 January 1996). Although miR-223 expression was dependent on the X-chromosome, the physiological or pathological expression of miR-223 was not different (*P*>0.05) among the C57BL/6J background mice or ARDS subjects.

### Construction of the BM chimeric mouse model

BM chimeric mice were constructed using the method described by Zhou *et al.*^52^ Briefly, following exposure to an X-ray dose of 5 Gy × 2, miRNA223−/+ and WT-C57BL/6J mice received an intravenous administration of BM cells (approximately 4 × 10^6^ cells) from miRNA223−/+ or WT-C57BL/6J mice not exposed to X-ray. The mice were examined 3 weeks after transplantation.

### Preparation of MTD-induced lung injury mice

Mitochondrial granules were isolated from C57BL/6J mouse muscle tissue using a Beyotime Biotechnology Kit (Beyotime Biotechnology, Shanghai, China) and suspended in 1 ml of Hank’s balanced salt solution buffer per 10 g of tissue. After the addition of protease inhibitor (1 : 100), the tissues were exposed to ultrasonic vibration. The samples were centrifuged at 12 000 × *g* for 10 min at 4 °C followed by centrifugation at 100 000 × *g* for 30 min; then the supernatant, which contained the MTDs, was collected. A BCA protein assay (Beyotime Biotechnology) was used to determine the protein concentration of the MTD solution. We used a 10 mg/kg intravenous injection of MTDs to induce lung injury in mice. The entire MTDs preparation process was sterile. Limulus reagent (A&C Biological Ltd, Zhangjiang, China) was used to detect endotoxin levels (<0.015 EU/ml). PCR was used to detect nuclear DNA content (<0.01%).

The miRNA223−/+ mice and WT mice were divided into 7 groups (12 mice in each group). At 6, 12, 24, 48, 72 and 96 h after intravenous injection of 10 mg/kg MTDs, the mice were fixed. PaO_2_/FiO_2_ levels were detected in the 0–72-h group. At the same time points, BALF was collected from half of the mice, and the other half was used to measure the lung dry/wet weight ratio. For the 96-h group of mice, all lung tissue was used for haematoxylin and eosin (HE) staining (Solarbio Ltd, Beijing, China).

### PaO_2_/FiO_2_, inflammatory cell counts in the BALF, the lung dry/wet ratio and lung injury pathological examination

PaO_2_/FiO_2_ in mice was measured using an ABL800 blood gas analyser (Radiometer, Copenhagen, Denmark).

After the mice were killed, the lung tissue was stripped, and the surface water was rinsed with physiological saline. The wet weight was measured, and then the lung tissues were dried at a constant temperature of 80 °C for 24 h. The dry weight was measured, and the lung dry/wet weight ratio was calculated.

The necks of the mice were cut, and the tissues surrounding the trachea were removed. Using a blunt syringe needle, the trachea was punctured, and the nose and mouth of the mice were connected to a 5-ml collector. The entire bronchial bronchus was lavaged with prechilled phosphate-buffered saline for a total of three times. The neutrophils, lymphocytes and macrophages were pelleted from BALF by centrifugation for 5 min at 25 °C. The total cell count was obtained using a 100 × oil objective in Wright’s solution (Sigma-Aldrich, Darmstadt, Germany).

The mice were fixed under deep anaesthesia; 4 °C prechilled heparin saline was used to irrigate the entire pulmonary circulatory system, removing the red blood cells after perfusion with 4% paraformaldehyde for internal fixation. All lung tissue was removed and fixed in 4% paraformaldehyde solution for 24 h. Then the tissue was paraffin embedded, and sections were acquired and stained with HE for histopathological observation (400 ×, Olympus, Tokyo, Japan).

### miRNA detection

The relative quantity of 29 inflammation-related miRNAs was determined by Genepharma (Shanghai, China) using their small-scale prescreening services. For the detection of miR-223, total miRNA was extracted from the BALF using the RNAeasy Small RNA Isolation Kit (Beyotime Biotechnology). Reverse transcription of miR-223 using a specific stem-loop primer (GenePharma) was performed using qPCR with a TaqMan miRNA Assay Kit (Applied Biosystems, Waltham, MA, USA). U6 was used as an internal control. The miR-223 reverse transcription primer was as follows: 5′-CTCAACTGGTGTCGTGGAGTCGGCAATTCAGTTGAG AACTGTCA-3′. The miR-223 quantitative primers were as follows: forward primer 5′-ACACTCCAGCTGGGACCCCATAAACTGTTT-3′ and reverse primer: 5′-TGGTGTCGTGGAGTCG-3′. The U6 primers were as follows: 5′-CTCGCTTCGGCAGCACA-3′ and 5′-AACGCTTCACGAATTTGCGT-3′.

### Detection of inflammatory cytokines

Inflammatory cytokine and chemokines were screened using the mouse Inflammation Array G1 (Raybiotech Ltd, Guangzhou, China) antibody chip according to the manufacturer’s instructions (≥1.5-fold increase or a decrease ≤0.65-fold indicates a significant difference). The sequences of the corresponding inflammatory cytokines are shown in Supplementary Figure S3A. The detection of IL-1*β* was performed using an ELISA kit (R&D Systems Inc., Minneapolis, MN, USA) according to manufacturer’s instructions.

### Isolation, purification and labelling of mouse BMDNs

BMDNs were obtained following the method described by Swamydas and Lionakis^53^ Briefly, the miRNA223−/+ or WT-C57BL/6J mice were sterilized, and the femurs and tibia were isolated and placed in a petri dish with ice-cold RPMI 1640 1 × containing 10% FBS and 1% penicillin/streptomycin. After cutting the bones, a syringe containing 10 ml of RPMI supplemented with 10% FBS and 2 mM EDTA was used to rinse the femoral/tibial pair. The solution was passed through a 100-*μ*m filter. In addition, the sample was centrifuged at 1400 × *g* for 7 min at 4 °C. Approximately 800 000 BM cells were harvested from each uninfected 8–12-week-old C57BL/6J mouse. Neutrophils were obtained by density gradient centrifugation. Neutrophils were labelled with CD45 (clone 30-F11), Ly6G (clone 1A8) and CD11b (clone M1/70) (Abcam, Cambridge, UK), and they were sorted by flow cytometry for subsequent experiments.

### Flow cytometry

Mouse peripheral blood, BALF and BM were centrifuged to remove cell debris and filtered through a 100-*μ*m filter to prepare a single-cell suspension. After adding 10 *μ*l of specific fluorescent monoclonal antibody, the cells were stained in the dark for 15 min. Flow cytometry was used to detect and sort the cell subsets. MHC II (M5/114.15.2), CD11b (M1/70), CD11c (N418), Ly6G (1A8), Ly6C (HK1.4), Sca-1 (D7), CD117 (ACK2), CD34 (RAM34) (Abcam) or the corresponding fluorescent dye-labelled streptavidin antibody was added to the cells. Cell analysis was performed using Attune NxT flow cytometry (Thermo Fisher, Waltham, MA, USA).

### RRPL3 3′UTR, miR-223 mimics and miR-223 inhibitor transfection

The pGL3-luc-NLRP3 3′UTR luciferase reporter gene was constructed using the miRNA-binding target prediction website (http://www.targetscan.org/) to predict the possibility of miRNA-223 binding to the NLRP3 3′UTR. PGL3-luc-NLRP3 3′UTR plasmid, pGL3-luc-NLRP3 3′UTRdelete plasmid, miR-223 mimics and miR-223 inhibitor were obtained from GenePharma. For cell transfections, 1 × 10^6^ or 10^7^ Ly6G+ BMDN were seeded in 96- or 12-well plates, and plasmids and miRNAs were transfected using Lipofectamine 3000 (Thermo Fisher) according to the instruction manual. A multifunctional fluoroscopy unit (Molecular Devices, Sunnyvale, CA, USA) was used to detect luciferase activity.

### miR-223 administration to mice *in vivo*

MCC950 and anakinra were purchased from MedChemExpress (Princeton, NJ, USA) and administered intraperitoneally at 40 mg/kg/day to mice. miR-223 was administered as the synthesized mmu-miR-223 via a nanoparticle. MaxSuppressor *In Vivo* RNA-LANCEr II (NLE) (Bioscientific, Austin, TX, USA) was used according to the manufacturer’s instructions to administer 100 *μ*g/kg/day mmu-miR-22 mimics intravenously.

### PAR-CLIP analysis

The enrichment of miR-223 in RISC was detected using the method described by Llobet-Navas *et al.*^54^ RISC consists of the Dicer enzyme, Argonaute protein, siRNA and other biological macromolecules. Briefly, the cells were pretreated with 10 *μ*M 4-thioureidine (Sigma-Aldrich) and irradiated with UV (200 mJ/cm^2^) at 4 °C overnight. The cells were then lysed on ice for 20 min. Then the cells were centrifuged at 12 000 × *g* for 15 min, and the cell pellet was resuspended and immunoprecipitated overnight at 4 °C using G beads (Roche, Basel, Switzerland) and 10 *μ*l of anti-AGO2 antibody (Cell Signaling Technology Inc., Boston, MA, USA). The expression level of NRPL3 mRNA in the final precipitate was examined by qPCR, and GAPDH, B2M and RPL13A (Cell Signaling Technology) were used as internal reference genes.

### Western blotting

The same amount of cellular protein extract (20 *μ*l) from each sample was separated and transferred to a nitrocellulose membrane (Beyotime Biotechnology) using 12% SDS-PAGE (Bio-Rad Inc., Hercules, CA, USA). Each membrane was incubated overnight at 4 °C with an antibody against NRPL3 (Abcam). The membrane was then incubated with the appropriate fluorescence-conjugated secondary antibody (LI-COR, Lincoln, NE, USA) at room temperature for 2 h. Imaging was performed using the Odyssey Imaging System (LI-COR).

### Statistical analysis

All data are shown as the mean±S.E.M. The data were analysed using Student’s *t*-test (*P<0.05 and **P<0.01). Statistical analyses and the plotting of the data were performed using GraphPad Prism 6.0 (GraphPad Software Inc., La Jolla, USA).

## Publisher’s Note

Publisher’s Note: Springer Nature remains neutral with regard to jurisdictional claims in published maps and institutional affiliations.

## Figures and Tables

**Table 1:** Demographics and clinical details of ARDS patient population

**Phenotype**	***n***	**Age**	**Pathogenic factors**	**Time to sample (day)**	**Relative miR-223 expression**	**Serum IL-1*****β*** **(pg/ml)**	**Lung Ly6G+ neutrophil (%)**	**PaO**_**2**_**/FiO**_**2**_ **(mm Hg)**	**ΔPaO**_**2**_**/FiO**_**2**_ **(mm Hg)**
Healthy controls	8	43.3	NA	2.5	18.92±4.1	12.08±3.31	5.49±0.54	455.13±23.15	−4.88±35.04
				6.3	19.21±3.6	11.56±6.62	5.81±0.61	450.25±27.54	
ARDS	26	46.9	Primary pneumonia	2.6	27.47±13.51	44.96±12.22	9.42±1.39	87.85±36.89	134.27±64.91
				7.2	18.54±8.56	36.4±8.15	6.11±0.41	222.12±55.29	
	17	41.8	Trauma	2.5	39.18±12.07	49.87±16.92	10.11±1.4	107.12±37.48	246.53±63.65
				6.6	35.56±6.44	19.22±7.28	6.45±0.89	353.65±71.35	
	7	39.7	Blood transfusion	3.1	38.95±13.98	37.92±6.85	9.83±1.6	88.8±26.20	345.6±26.87
				6.8	45.15±14.6	13.45±3.59	4.89±1.44	434.4±29.7	
	5	33.2	Aspiration	3.3	17.4±5.6	51.91±10.31	9.92±1.54	112.14±24.88	147.14±56.49
				7.2	20.11±9.54	49.57±8.78	8.56±0.78	259.26±51.08	

Values represent mean±S.D.
