# Supplementary material for: Ly6G+ neutrophil-derived miR-223 inhibits the NLRP3 inflammasome in mitochondrial DAMP-induced acute lung injury
Source: Cell Death Dis. 2017 Nov 16;8(11):e3170–. doi: 10.1038/cddis.2017.549 (PMC5775410; doi:10.1038/cddis.2017.549)
Supplement: Supplementary Figure Legends [file cddis2017549x1.docx]

**Supplementary Information:**

**Figure. S1. Expression of immune-related miRNA in the BALF of ARDS patients with different aetiologies.**

BALF was collected from 63 patients with ARDS and healthy controls and was classified based on different aetiologies, including infection, trauma, blood transfusion and inhalation. The relative expression of 29 inflammation-associated miRNAs was pre-screened using a small-scale miRNA microarray chip to show miRNA expression levels relative to an endogenous control, sno202. The expression levels were then normalized using median normalization (A). Data are presented in the form of a heatmap showing log2-transformed relative expression (DCt). Red fluorescence indicated downregulated expression, and green fluorescence indicated upregulated expression.

The linear relationship of has-miR-223 (B), LY6G+ neutrophil (D) and IL-1β (C) levels with prognostic effect (ΔPaO2/FiO2) was analysed in samples from acute-phase ARDS patients after 4 days of PEEP. Similarly, the association of has-miR-223 with LY6G+ neutrophil (E) and IL-1β (F) levels in BALF was also analysed. The linear relationship fitting curve formula is Y=YIntercept+Slope*X, where YIntercept=(initial value to be fit); and Slope=[(YMAX-YMIN)/(XMAX-XMIN)]. The relative expression level of has-miR-223 in the BALF from 63 healthy subjects and ARDS patients in the acute phase were compared based on sex. The expression level of mmu-miR-223 was statically and physiologically similar 25 C57BL/6J background mice based on sex (G). All data are presented as the mean±SEM, and comparisons between groups were performed using t-tests.

**Figure. S2. The proportion of pulmonary myeloid cells in ARDS patients.**

BALF was collected from 63 patients with ARDS and healthy controls and was classified based on different aetiologies, including infection, trauma, blood transfusion and inhalation. After labelling with specific antibodies, including MHCII, CD11b, LY6C, LY6G and CD11c, flow cytometry was performed to identify neutrophils (Ly6G+ MHCII−) (A), monocytes (Ly6C+ MHCII-), intermediate monocytes (Ly6C+ MHCII+), macrophages (Ly6C- MHCII+) and dendritic cells (MHCII+ CD11c+ CD11b-) during the acute period and remission period (B). All data are presented as the mean±SEM, and comparisons between groups were performed using t-tests. *P<0.05 and **P<0.01; n=5-26.

**Figure. S3. Mouse inflammation antibody array G-Series 1 map.**
