# Supplementary figures and images for: Ly6G+ neutrophil-derived miR-223 inhibits the NLRP3 inflammasome in mitochondrial DAMP-induced acute lung injury
Source: Cell Death Dis. 2017 Nov 16;8(11):e3170–. doi: 10.1038/cddis.2017.549 (PMC5775410; doi:10.1038/cddis.2017.549)

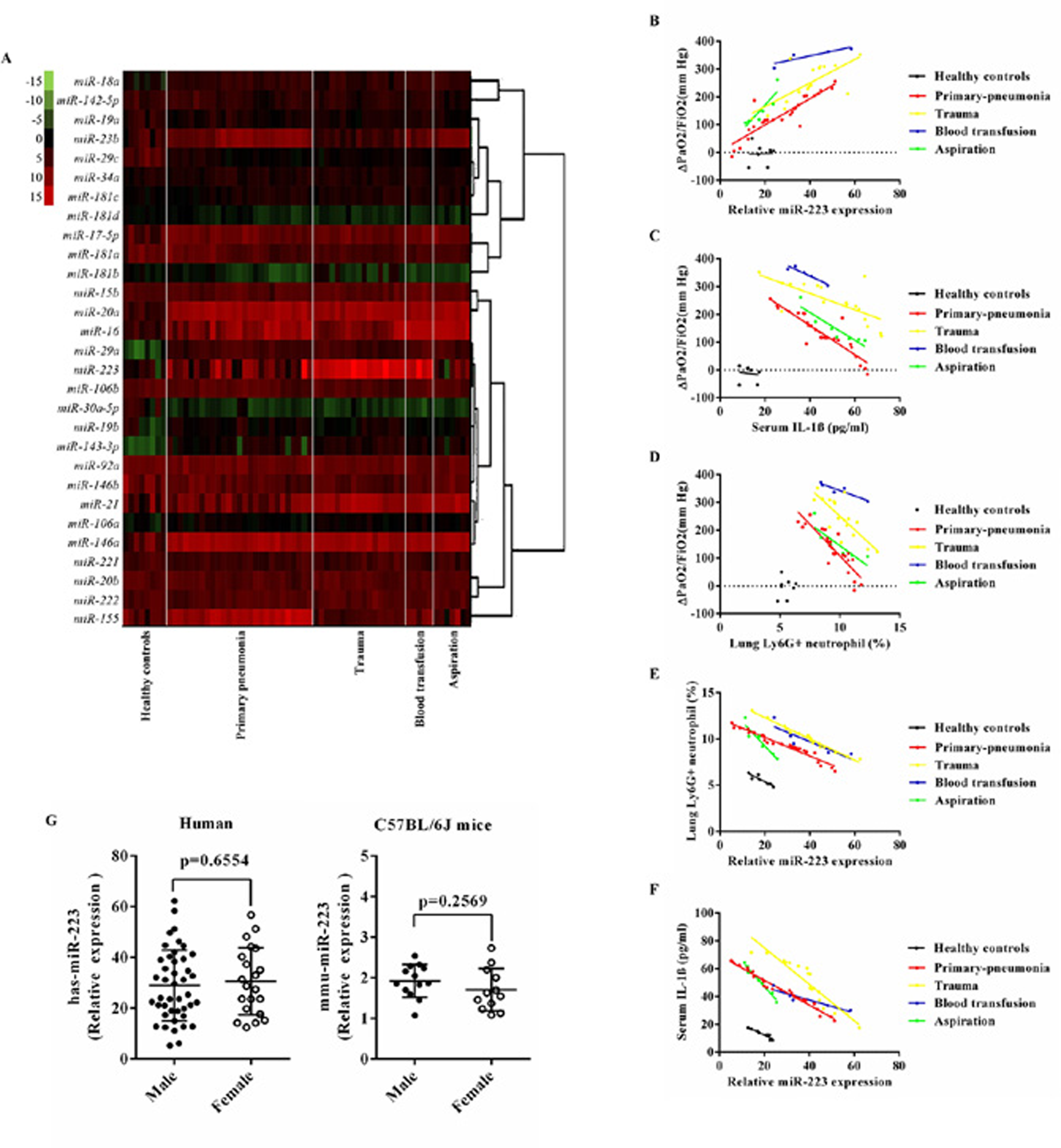

Supplement: Supplementary Figure S1 [file cddis2017549x2.tif]

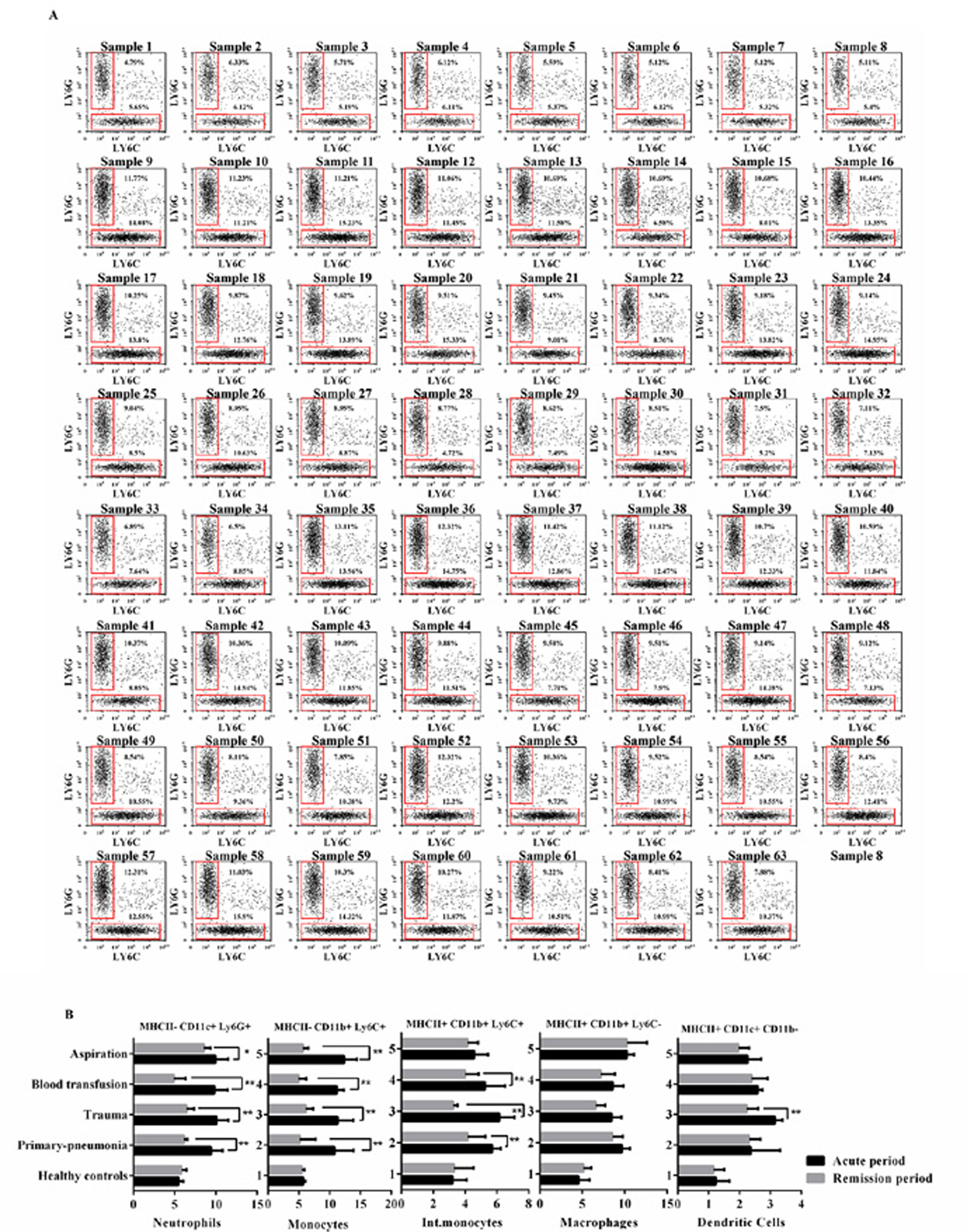

Supplement: Supplementary Figure S2 [file cddis2017549x3.tif]

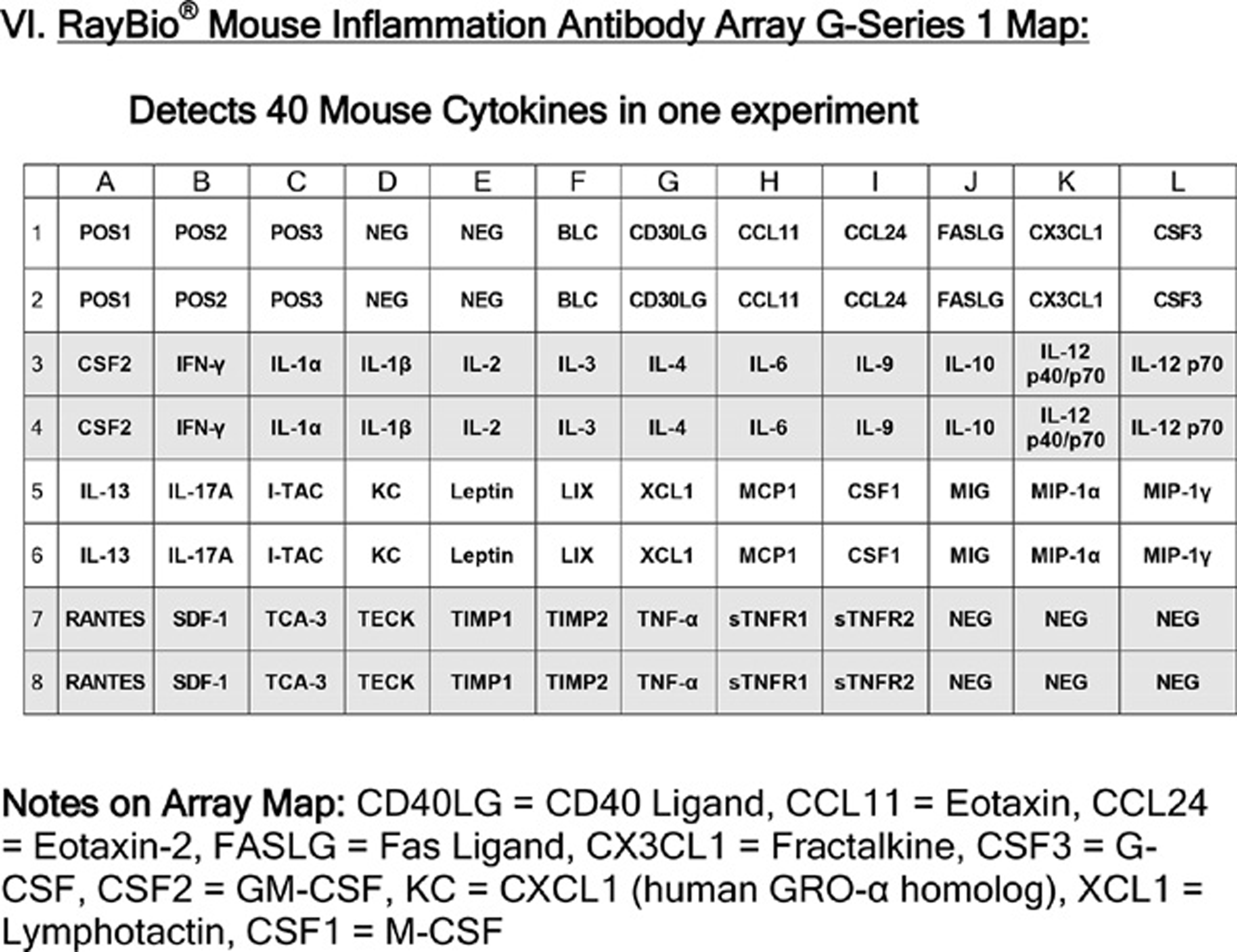

Supplement: Supplementary Figure S3 [file cddis2017549x4.tif]
